# Intravascular haemolysis in severe *Plasmodium knowlesi* malaria: association with endothelial activation, microvascular dysfunction, and acute kidney injury

**DOI:** 10.1038/s41426-018-0105-2

**Published:** 2018-06-06

**Authors:** Bridget E. Barber, Matthew J. Grigg, Kim A. Piera, Timothy William, Daniel J. Cooper, Katherine Plewes, Arjen M. Dondorp, Tsin W. Yeo, Nicholas M. Anstey

**Affiliations:** 1 Global and Tropical Health Division, Menzies School of Health Research, Darwin, NT Australia; 2 Infectious Diseases Society Sabah-Menzies School of Health Research Clinical Research Unit, Kota Kinabalu, Sabah Malaysia; 3 Jesselton Medical Centre, Kota Kinabalu, Sabah Malaysia; 4 Clinical Research Centre, Queen Elizabeth Hospital, Kota Kinabalu, Sabah Malaysia; 5 Mahidol Oxford Tropical Medicine Research Unit, Mahidol University, Bangkok, Thailand; 6 Department of Medicine, University of British Columbia, Vancouver, BC Canada; 7 Centre for Tropical Medicine and Global Health, Nuffield Department of Clinical Medicine, University of Oxford, Oxford, UK; 8 Lee Kong Chian School of Medicine, Nanyang Technological University, Singapore, Singapore; 9 Institute of Infectious Disease and Epidemiology, Tan Tock Seng Hospital, Singapore, Singapore

## Abstract

*Plasmodium knowlesi* occurs throughout Southeast Asia, and is the most common cause of human malaria in Malaysia. Severe disease in humans is characterised by high parasite biomass, reduced red blood cell deformability, endothelial activation and microvascular dysfunction. However, the roles of intravascular haemolysis and nitric oxide (NO)-dependent endothelial dysfunction, important features of severe falciparum malaria, have not been evaluated, nor their role in acute kidney injury (AKI). In hospitalised Malaysian adults with severe (*n* = 48) and non-severe (*n* = 154) knowlesi malaria, and in healthy controls (*n* = 50), we measured cell-free haemoglobin (CFHb) and assessed associations with the endothelial Weibel–Palade body (WPB) constituents, angiopoietin-2 and osteoprotegerin, endothelial and microvascular function, and other markers of disease severity. CFHb was increased in knowlesi malaria in proportion to disease severity, and to a greater extent than previously reported in severe falciparum malaria patients from the same study cohort. In knowlesi malaria, CFHb was associated with parasitaemia, and independently associated with angiopoietin-2 and osteoprotegerin. As with angiopoietin-2, osteoprotegerin was increased in proportion to disease severity, and independently associated with severity markers including creatinine, lactate, interleukin-6, endothelial cell adhesion molecules ICAM-1 and E-selectin, and impaired microvascular reactivity. Osteoprotegerin was also independently associated with NO-dependent endothelial dysfunction. AKI was found in 88% of those with severe knowlesi malaria. Angiopoietin-2 and osteoprotegerin were both independent risk factors for acute kidney injury. Our findings suggest that haemolysis-mediated endothelial activation and release of WPB constituents is likely a key contributor to end-organ dysfunction, including AKI, in severe knowlesi malaria.

## Introduction

The monkey parasite *Plasmodium knowlesi* is an important emerging zoonotic infection in Southeast Asia, and is now the most common cause of human malaria in Malaysia^1, 2^ and regions of western Indonesia^3, 4^. The risk of severe disease in adults is at least as high as in falciparum malaria^5, 6^, and fatal cases occur^1, 7^. Features of severe knowlesi malaria are similar to those of severe falciparum malaria in adults, and include hyperparasitaemia, jaundice, acute kidney injury (AKI), respiratory distress, shock and metabolic acidosis^5, 6^. However, in contrast to *P. falciparum*, *P. knowlesi*-attributed coma has not been reported to-date, and endothelial cytoadherence, a key pathogenic feature of severe falciparum malaria, does not appear to occur^8, 9^. Thus, alternative pathogenic mechanisms may play a greater role in severe knowlesi malaria. We have recently reported that, as with falciparum malaria, disease severity in knowlesi malaria is associated with parasite biomass, endothelial activation, and microvascular dysfunction^10^, as well as reduced red blood cell (RBC) deformability^11^. However, the roles of intravascular haemolysis and nitric oxide (NO)-dependent endothelial dysfunction, important features of severe falciparum malaria^12–16^, have not yet been reported.

In conditions associated with intravascular haemolysis, such as severe falciparum malaria, the cell-free haemoglobin (CFHb) released during erythrocyte rupture is readily oxidised from ferrous (Fe^2+^) to ferric (Fe^3+^) haemoglobin. Ferric haemoglobin (methaemoglobin) then releases haem^17^, which due to its hydrophobic nature readily intercalates into cell membranes and increases susceptibility to oxidant-mediate damage^18^. Free haem mediates a range of other pathogenic effects, including increased production of reactive oxygen species and proinflammatory cytokines^19, 20^, and upregulation of endothelial cell adhesion molecules^21^. CFHb is also able to quench nitric oxide (NO), and in adults with falciparum malaria is associated with reduced NO-dependent endothelial function, and hyperlactatemia, suggesting a role in impaired tissue perfusion^12^.

CFHb has also been shown to stimulate degranulation of endothelial Weibel–Palade bodies (WPBs) via TLR4 signalling^22, 23^. WPBs are storage organelles specific to endothelial cells, and upon endothelial activation, fuse with endothelial cell membranes and release their contents into plasma. Thus, constituents of WPBs, including von Willebrand factor (vWF), angiopoietin-2, P-selectin, and osteoprotegerin (OPG), are considered key markers and mediators of endothelial activation. Plasma concentrations of vWF have been shown to increase soon after inoculation of *P. falciparum* into human volunteers^24^, and both vWF and OPG increase early in *P. berghei*-infected mice^25, 26^, suggesting that endothelial activation is an early host response in malaria. In both knowlesi and falciparum malaria, endothelial activation is associated with disease severity and measures of impaired organ perfusion^10, 27^.

In severe knowlesi malaria, high parasitaemias can develop rapidly, and intravascular haemolysis has been reported^28^. However, the contribution of this process to disease severity, and the association with endothelial activation and dysfunction, has not been evaluated. We therefore measured CFHb in Malaysian adults with severe and non-severe knowlesi malaria, and assessed associations with the endothelial WPB constituents angiopoietin-2 and osteoprotegerin, endothelial and microvascular function, and other markers of disease severity.

## Results

### Patients

A total of 202 patients with knowlesi malaria were enroled, including 154 with non-severe and 48 with severe malaria by WHO criteria^29^, in addition to 50 healthy controls. Clinical and pathophysiological data from a subset of these patients have been previously reported^5, 10^. Baseline demographic, clinical and laboratory features of patients and controls are shown in Table 1.

**Table 1:** Baseline characteristics of patients with knowlesi malaria and healthy controls

Variable	Controls (*n* = 50)	Non-severe knowlesi malaria (*n* = 154)	Severe knowlesi malaria (*n* = 48)	*P* value (severe vs. NS knowlesi malaria)
Age	35 (22–43)	40 (28–53)	55 (47–62)	<0.0001
Male sex, *n* (%)	34 (68)	122 (79)	35 (73)	NS
Fever duration, days	NA	5 (4–7)	6 (3–7)	NS
Time from malaria treatment to enrolment, h	NA	5.8 (0–11.8)	6.5 (0–12.1)	NS
Parasites/µL	NA	3781 (979–13,680)	98,974 (24,034–164,304)	<0.0001
Haemoglobin, g/dL, mean (SD)	NA	12.9 (1.5)	11.9 (2.1)	0.0002
Haemoglobin nadir, g/dL, mean (SD)	NA	11.7 (1.5)	9.4 (2.0)	<0.0001
Haemoglobin fall, g/dL	NA	1.2 (0.5–1.8)	2.2 (1.6–3.2)	<0.0001
Cell-free haemoglobin, ng/mL	15,146 (9641–25,256)	37,568 (17,168–55,251)	67,923 (29,292–163,848)	<0.0001
Haptoglobin, g/dL	1.44 (1.01–1.72)	0.30 (0.07–1.18) *N* = 99	0.11 (0.04–0.21) *N* = 47	0.004
Platelets, ×10^3^/µL	NA	51 (36–76)	31 (21–57)	0.0001
Creatinine, µmol/L	NA	95 (77–113)	144 (112–207)	<0.0001
Bilirubin, µmol/L	NA	17 (13–25)	39 (24–88)	<0.0001
Aspartate transaminase, IU/L	NA	40 (29–52)	58 (39–103)	<0.0001
Alanine transaminase, IU/L	NA	40 (24–62)	37 (21–57)	NS
Lactate, mmol/L	NA	1.2 (0.9–1.5) *N* = 134	1.5 (1.1–2.3)	0.0001
Interleukin-6, pg/mL	BDL 27/30	38 (18–83) *N* = 97	182 (56–353) *N* = 47	<0.0001
WBP constituents				
Angiopoietin-2, pg/mL	1,183 (875–1597)	4,296 (2943–6323)	10,072 (6311–14,072)	<0.0001
P-selectin, pg/mL	40 (31–52)	31 (25–39) *N* = 153	39 (30–51) *N* = 46	0.0008
Osteoprotegerin, pg/mL	986 (625–1463)	2087 (1605–3008) *N* = 153	4795 (3184–7535) *N* = 46	<0.0001
vWF, pg/mL	1156 (843–1634)	5328 (3952–6188) *N* = 38	5140 (4555–6336) *N* = 47	NS
Adhesion molecules				
ICAM-1, pg/mL	149 (123–167)	469 (363–621)	563 (430–703)	0.004
E-selectin, pg/mL	19 (13–25)	49 (36–66)	63 (50–90)	0.0003
Microvascular reactivity, units/s	6.62 (5.43–7.25) *N* = 43	6.1 (5.3–6.9) *N* = 59	3.5 (2.8–5.3) *N* = 41	<0.0001
Endothelial function (RHPAT)	1.97 (1.7–2.27)	1.87 (1.59–2.23) *N* = 64	1.47 (1.33–1.79) *N* = 38	<0.0001

Data are median (IQR) unless otherwise stated

*NS* non-severe, *NA* not assessed, *BDL* below detection limit, *WPB* Weibel–Palade body, *vWF* von Willebrand factor, *ICAM-1* intercellular adhesion molecule-1, *RHPAT* reactive-hyperaemia peripheral arterial tonometry

Among the 48 patients with severe knowlesi malaria, WHO severity criteria included hyperparasitaemia (*n* = 24, 50%), jaundice (*n* = 21, 44%), respiratory distress (*n* = 14, 29%), severe AKI by WHO criteria (Cr > 265 mmol/L; *n* = 11, 23%), shock (*n* = 11, 23%), metabolic acidosis (*n* = 4, 8%), severe anaemia (*n* = 5, 10%) and abnormal bleeding (*n* = 5, 8%). Nineteen patients (40%) had one severity criterion, 17 (35%) had two and 12 (25%) had three or more. Using KDIGO criteria to define AKI (and the MDRD equation to estimate baseline creatinine, see 'Materials and Methods'), AKI was present on admission in 40 (83%) patients with severe malaria and 44 (29%) patients with non-severe malaria. AKI developed during admission in another two (4%) patients with severe and two (1%) patients with non-severe malaria.

### Intravascular haemolysis

**Fig. 1:**
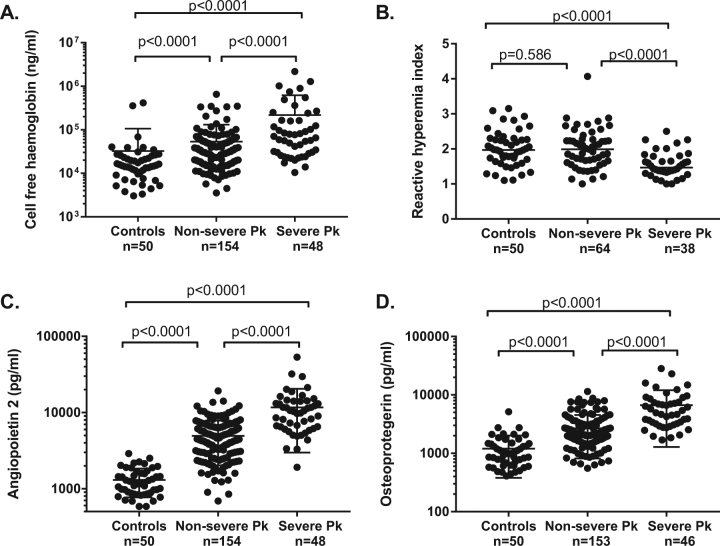
Cell-free haemoglobin (**a**), endothelial function (**b**), angiopoietin-2 (**c**) and osteoprotegerin (**d**) in patients with severe and non-severe knowlesi malaria, and healthy controls

CFHb was significantly higher in patients with severe and non-severe knowlesi malaria compared to controls (67,923 ng/mL, 37,568 ng/mL and 15,146 ng/mL, respectively, *p* < 0.0001 for both comparisons), and higher in those with severe compared to non-severe disease (*p* < 0.0001) (Table 1 and Fig. 1). CFHb was higher in patients with severe knowlesi malaria compared to patients with severe falciparum malaria from the same cohort (69,923 ng/mL vs. 35,322 ng/mL [*n* = 21; data previously published^30^], *p* = 0.015). Haptoglobin was lower in patients with severe and non-severe knowlesi malaria compared to controls (0.11 g/dL, 0.30 g/dL, and 1.44 g/dL, respectively, *p* < 0.0001 for both comparisons), and lower in patients with severe compared to non-severe knowlesi malaria (*p* = 0.004). In patients with severe and non-severe knowlesi malaria, there was no significant difference in CFHb or haptoglobin in those enrolled prior to, compared to post, commencement of antimalarial treatment (Supplementary Table 1).

### Endothelial and microvascular function

Endothelial function, as measured by the reactive-hyperaemia index (RHI), was lower in patients with severe knowlesi malaria compared to those with non-severe knowlesi malaria (median RHI 1.47 vs. 1.87, *p* < 0.0001; Table 1). However, there was no significant difference between controls and patients with non-severe knowlesi malaria. In patients with severe knowlesi malaria, endothelial function was at least as low as it was in patients with severe falciparum malaria from the same cohort (1.47 vs. 1.53 [1.26–1.75], *n* = 17, *p* = 0.778; previously published^30^). Endothelial function was associated with *P. knowlesi* parasitaemia (*r* = −0.20, *p* = 0.044), and with lactate (−0.24, *p* = 0.024), with the latter remaining significant after controlling for parasitaemia (*p* = 0.041). There was no association between endothelial function and CFHb.

Microvascular function, as measured by NIRS, was reduced in patients with knowlesi malaria in proportion to disease severity (Table 1, and as previously reported^10^). There was a positive correlation between endothelial and microvascular function as measured by RHI and NIRS, respectively (*r* = 0.35, *p* <0.001), remaining significant after controlling for parasitaemia and age (*r* = 0.244, *p* = 0.020).

### Cell-free haemoglobin and markers of disease severity

CFHb was correlated with parasitaemia (*r* = 0.24, *p*<0.001) (Table 2). CFHb also correlated with lactate (*r* = 0.20, *p* = 0.006), and with microvascular dysfunction (*r* = 0.35, *p* <0.001), with both correlations remaining significant after controlling for parasitaemia (*p* = 0.040 and 0.028, respectively). CFHb correlated with creatinine (0.31, *p* < 0.0001), remaining significant after controlling for parasitaemia (*p* = 0.001). Using logistic regression and controlling for parasitaemia, log CFHb was associated with risk of AKI by KDIGO criteria on or during admission (OR 1.52 [95% CI: 1.12–2.07], *p* = 0.008). There was no association between CFHb and patient age.

**Table 2:** Cell-free haemoglobin and correlations with markers of severity in knowlesi malaria

	Univariate analysis		Controlling for parasitaemia	
	Correlation coefficient	*P* value	Correlation coefficient	*P* value
Parasite count	0.23	<0.001		
Creatinine	0.31	<0.0001	0.23	0.001
Lactate	0.20	0.006	0.15	0.040
AST	0.37	<0.0001	0.35	<0.0001
ALT	0.09	0.222	NA	
IL-6	0.28	<0.001	0.23	0.001
Microvascular reactivity	−0.35	<0.001	−0.22	0.028
Angiopoietin-2	0.33	<0.0001	0.24	0.001
OPG	0.37	<0.0001	0.34	<0.0001
ICAM-1	0.17	0.019	0.12	0.081
E-selectin	0.28	<0.0001	0.18	0.010

Univariate correlations were calculated using Spearman’s correlation coefficient. Partial correlation was used to control for parasitaemia, with all variables log-transformed. Correlations with parasitaemia, OPG, AST and IL-6 all remained significant after also controlling for angiopoietin-2. No association was found between cell-free haemoglobin and the Weibel–Palade body (WBP) constituents P-selectin or vWF

*AST* aspartate transaminase, *ALT* alanine transaminase, *IL* interleukin, *OPG* osteoprotegerin, *ICAM-1* intercellular adhesion molecule, *NA* not assessed

CFHb was correlated with aspartate transaminase (*r* = 0.38, *p* < 0.0001), likely reflecting release of this enzyme from RBCs^31^. There was no association between CFHb and the other liver aminotransaminase, alanine transaminase.

### Cell-free haemoglobin and association with WPB constituents

As CFHb has been shown to stimulate degranulation of WPBs^22, 23^, we evaluated plasma concentrations of the WPB constituents angiopoietin-2, OPG, P-selectin, and VWF, and their associations with CFHb. Angiopoietin-2 was increased in patients with knowlesi malaria compared to controls, and increased in severe compared to non-severe disease (Table 1, Fig. 1 and as previously reported in a subset of these patients^10^). Similarly, OPG was increased in severe compared to non-severe knowlesi malaria (median 4795 vs. 2087 pg/mL, *p* < 0.0001), and increased in both groups compared to controls (*p* < 0.0001 for both comparisons). No increase in P-selectin was seen in knowlesi malaria patients overall compared to controls, and VWF, although increased in knowlesi malaria patients compared to controls, was not increased in severe compared to non-severe disease (Table 1). OPG correlated with angiopoietin-2, after controlling for age and parasitaemia (*r* = 0.39, *p* < 0.0001).

CFHb correlated with angiopoietin-2 (*r* = 0.33, *p* < 0.0001) and OPG (*r* = 0.37, *p* < 0.0001), with both correlations remaining significant after controlling for parasitaemia (*p* = 0.0005 and *p* < 0.0001, respectively; Table 2). The association between CFHb and OPG remained significant after also controlling for angiopoietin-2.

Both angiopoietin-2 and OPG correlated with age in knowlesi malaria patients (*r* = 0.39, *p* < 0.0001, and *r* = 0.43, *p* < 0.0001, respectively), independent of parasitaemia. OPG also correlated with age in healthy controls (*r* = 0.30, *p* = 0.033). Age is a known risk factor for severe knowlesi malaria^10^. However, in a backward stepwise logistic regression model controlling for age and parasitaemia, both OPG and angiopoietin-2 (but not CFHb) remained as independent risk factors for severe malaria (with hyperparasitaemia removed as a severity criterion), and for AKI (Table 3; alternative logistic regression models shown in Supplementary Tables 2 and 3). vWF was also associated with risk of AKI on univariate analysis (odds ratio for log-transformed vWF 4.13 [95% CI: 1.07–15.98], *p* = 0.040); however, this did not remain significant after controlling for parasitaemia and age.

**Table 3:** Logistic regression model for predictors of acute kidney injury and severe malaria in knowlesi malaria

	Odds ratio	95% Confidence interval	*P* value
Predictors of AKI			
Log angiopoietin-2	4.41	2.02–9.63	<0.0001
Log osteoprotegerin	1.98	1.02–3.82	0.043
Age^a^	1.07	1.04–1.10	<0.0001
Predictor of severe malaria			
Log angiopoietin-2	5.35	2.01–14.25	0.001
Log osteoprotegerin	2.66	1.15–6.14	0.022
Log parasite count	1.47	1.15–1.89	0.002

*AKI* acute kidney injury as defined by KDIGO. Backward stepwise regression was used, with variables removed if *P* value was >0.05. Variables included in both models included: age, angiopoietin-2, osteoprotegerin, parasite count, and cell-free haemoglobin. Patients with hyperparasitaemia as a sole severity criterion were reclassified as non-severe for this analysis. Alternative regression models are included in Supplementary Data

^a^Age remained an independent risk factor of AKI if included as a binary variable of >45 years (OR 6.09 [95% CI: 2.91–12.77], *P* < 0.0001). For predictors of severe malaria, age was not an independent risk factor, whether included as a continuous variable, or as a binary variable of >45 years

### Osteoprotegerin and correlation with endothelial cell adhesion molecules and IL-6

As with angiopoietin-2 (Table 4, and as previously reported^10^), OPG was also associated with ICAM-1 (*r* = 0.31, *p* < 0.0001) and E-selectin (*r* = 0.34, *p* < 0.0001), with both correlations remaining significant after controlling for parasitaemia and age (*p* ≤ 0.0001 for both correlations). OPG was also correlated with IL-6 (*r* = 0.54, *p* < 0.0001), remaining significant after controlling for parasitaemia and age (*p* < 0.0001). The correlations between OPG and E-selectin, ICAM-1 and IL-6 were also independent of angiopoietin-2 (Table 4).

**Table 4:** Comparative correlations between Weibel–Palade body constituents OPG and angiopoietin-2 and biomarkers of severity in knowlesi malaria

	OPG				Angiopoietin-2			
	Univariate analysis		Controlling for parasitaemia and age		Univariate analysis		Controlling for parasitaemia and age	
	Correlation coefficient	*P* value	Correlation coefficient	*P* value	Correlation coefficient	*P* value	Correlation coefficient	*P* value
Age	0.43	<0.0001	0.32^a^	<0.0001	0.39	<0.0001	0.30^a^	<0.0001
Parasite count	0.45	<0.0001	0.38^b^	<0.0001	0.46	<0.0001	0.32^b^	<0.0001
Creatinine	0.40	<0.0001	0.36	<0.0001	0.54	<0.0001	0.54	<0.0001
Lactate	0.38	<0.0001	0.31	<0.0001	0.34	<0.0001	0.25	0.0009
AST	0.32	<0.0001	0.29	0.0001	0.30	<0.0001	0.29	0.0001
IL-6	0.57	<0.0001	0.34	0.0001	0.45	<0.0001	0.26	0.0002
Microvascular reactivity	−0.48	<0.0001	−0.23	0.024	−0.46	<0.0001	−0.22	0.029
Angiopoietin-2	0.52	<0.0001	0.39	<0.0001				
VWF	0.26	0.018	0.32	0.004	0.28	0.010	0.28	0.010
P-selectin	0.23	0.0009	0.14	0.039	0.18	0.010		NS
ICAM-1	0.31	<0.0001	0.27	0.0001	0.36	<0.0001	0.33	<0.0001
E-selectin	0.34	<0.0001	0.31	<0.0001	0.30	<0.0001	0.26	0.0002
RHPAT	−0.39	0.0001	0.26	0.011	−0.23	0.020		NS

All biomarkers of severity remained significantly correlated with OPG after also controlling for angiopoietin-2, except microvascular reactivity and ICAM-1. In contrast, after controlling for OPG, only creatinine, AST and ICAM-1 remained significantly associated with angiopoietin-2

^a^Controlling for parasitaemia only

^b^Controlling for age only

### Osteoprotegerin and markers of disease severity

In addition to the association with adhesion molecules and IL-6, after controlling for age and parasitaemia OPG was also independently correlated with all other malaria severity markers evaluated, including creatinine (*r* = 0.36, *p* < 0.0001), lactate (*r* = 0.31, *p* < 0.0001), microvascular dysfunction (*r* = 0.23, 0.024) and endothelial dysfunction (*r* = 0.26, *p* = 0.011) (Table 4). These associations were at least as strong as with the well-validated malaria severity biomarker angiopoietin-2, and, in the case of lactate, AST, creatinine, and endothelial dysfunction, were independent of angiopoietin-2 (Table 4).

## Discussion

Intravascular haemolysis (as measured by CFHb) is increased in knowlesi malaria in proportion to disease severity, and to a greater extent than that seen in falciparum malaria. Furthermore, intravascular haemolysis is independently associated with markers of disease severity, including lactate, microvascular dysfunction, and creatinine, suggesting that haemolysis likely contributes to impaired tissue perfusion and organ dysfunction in knowlesi malaria. With the apparent paucity of *P. falciparum*-like endothelial cytoadherence causing sequestration in knowlesi malaria, our findings suggest that intravascular haemolysis may play a more central role in the development of severe disease in knowlesi compared to falciparum malaria.

As with falciparum malaria, the cause of intravascular haemolysis in severe knowlesi malaria is likely multifactorial, with lysis of infected and uninfected red blood cells both contributing. The greater severity of intravascular haemolysis in severe knowlesi compared to *P. falciparum* may reflect the 24-h erythrocytic life-cycle of *P. knowlesi*, or may reflect poor adaption of *P. knowlesi* to the human host. Massive destruction of RBCs has been previously reported in *P. knowlesi*-infected rhesus macaques (*Macaca mulatta*), another unnatural host for this parasite. As well as the red cell agglutination and sludging reported in these early studies^32^, haemolytic phenomena such as haemoglobinuria, renal tubular acidosis and haemoglobin casts within tubular lumens were frequently noted as pre-terminal events^33, 34^. The marked haemolysis associated with *P. knowlesi* makes this parasite an ideal model to study the pathophysiological consequences of haemolysis in severe human malaria.

The mechanisms by which haemolysis mediates end-organ damage in severe malaria are incompletely understood. In severe falciparum malaria, haemolysis is associated with impaired NO-dependent endothelial dysfunction^12^, and with oxidative damage which contributes to AKI^15^. In addition, we now show that in knowlesi malaria, intravascular haemolysis is independently associated with the endothelial cell WPB constituents angiopoietin-2 and OPG, suggesting that endothelial activation is likely a key mediator of haemolysis-induced end-organ damage. An association between CFHb and angiopoietin-2 has been previously demonstrated in adults^12^ and children^13^ with falciparum malaria; however, we now extend these findings by demonstrating an association in *P. knowlesi* between CFHb and both angiopoietin-2 and OPG that is independent of parasite biomass. Our findings are consistent with previous in vitro and murine reports demonstrating that CFHb stimulates degranulation of WPBs^22, 23^. In the current study, the lack of independent associations with CFHb and markers of end-organ damage, after controlling for angiopoietin-2 and/or OPG, further supports the role of endothelial activation in mediating pathophysiological consequences of intravascular haemolysis.

While increased angiopoietin-2 is well documented in severe malaria, and known to be a key marker of disease severity^10, 27^, a notable finding of our study was the marked elevation of the other key WPB constituent, OPG, in severe knowlesi malaria. OPG is a member of the tumour necrosis factor (TNF) receptor superfamily, and is a soluble decoy receptor for the receptor activator of NF-кB ligand (RANKL), thus modulating the interaction between RANKL and its receptor RANK^35^. OPG has a widespread tissue distribution, including in vascular and immune tissues. In vascular tissues, release of OPG from endothelial cells is upregulated by cytokines including TNF, IL-1a and IL-1b^36, 37^. OPG has been shown to stimulate endothelial cell migration^38^, to increase leukocyte adhesion to endothelial cells both in vitro and in vivo^39^, and to upregulate endothelial cell adhesion molecules in the presence of TNF^40^. This latter effect of OPG is consistent with the results of our current study, with OPG independently associated with endothelial adhesion molecules ICAM-1 and E-selectin. Upregulation of adhesion molecules by OPG may also explain in part the association between OPG and mortality in a recent study of African children with cerebral malaria^25^.

We also found an independent association of OPG with endothelial and microvascular dysfunction, as measured by RHPAT and NIRS, respectively. This is consistent with other studies which have demonstrated an association between OPG and endothelial dysfunction in other conditions, including hyperuricemia^41^, Hashimoto’s thyroiditis^42^ and type 1 diabetes mellitus^43^. In addition, OPG is elevated in other conditions associated with endothelial dysfunction, such as cardiovascular disease, and in patients with diabetes mellitus is associated with adverse cardiovascular outcomes and mortality^44^. Endothelial dysfunction is a key feature of severe malaria, resulting from reduced NO bioavailability^16^. OPG is known to block RANKL-induced activation of the intracellular eNOS pathway in vitro, and to reduce endothelial NO production^45^. The association between OPG and endothelial dysfunction in severe knowlesi malaria suggests OPG likely exacerbates endothelial NO deficiency, contributing to severe disease. OPG inhibition of eNOS and endothelial NO production is reversed in vitro by RANKL^45^. Taken together, these findings raise the possibility that RANKL may be a candidate adjunctive treatment to improve NO bioavailability in conditions associated with elevated OPG and endothelial dysfunction such as severe malaria.

In addition to its release from endothelial cells, OPG is also expressed in immune cells, including dendritic cells and macrophages, and may modulate inflammatory responses through inhibition of RANKL/RANK signalling^46^. As RANKL has been shown to reduce macrophage production of proinflammatory cytokines^47^, inhibition of RANKL by OPG may be expected to increase inflammatory responses. In keeping with this, in our study, OPG was independently associated with IL-6. This is also consistent with a murine study, in which inflammatory cytokines, including IL-6, TNF, IL-1B and MCP-1, were reduced in OPG knockout mice and in WT mice infused with RANKL^46^.

In this study we found that OPG and angiopoietin-2 were both independently associated with AKI, suggesting that haemolysis-induced endothelial activation is an important mechanism of malaria-associated AKI. AKI is common in knowlesi malaria, occurring in 44% of all patients in this study, and in 88% of those with severe disease. AKI is now recognised to have significant long-term consequences, including increased risk of chronic kidney disease, cardiovascular disease and death (reviewed in ref. ^48^), and new treatment strategies to prevent malaria-associated AKI are needed. In falciparum malaria, haemolysis has been linked to AKI from oxidative stress and lipid peroxidation^49^, and further studies are warranted to determine if haemolysis-induced oxidative stress also contributes to AKI in knowlesi malaria. The pathogenic pathways of CFHb may present targets for adjunctive treatments to protect against AKI in both falciparum and knowlesi malaria^50, 51^.

Our study is associated with several limitations. First, although our findings suggest that haemolysis-induced endothelial activation and WPB release may be key pathogenic mechanisms in severe malaria, it is possible that release of WPB constituents may also occur through alternate mechanisms, such as direct effect of parasite products, or cytokines induced at schizogony^52^. Parasite products may directly stimulate endothelial cells and have been implicated in WBP exocytosis in falciparum malaria^27^. Interestingly, the related parasite *Cryptosporidium* has been shown to upregulate OPG mRNA in intestinal epithelial cells, with the increase in OPG serving as an anti-apoptotic and parasite survival strategy^53^. Nevertheless, in our study the association between CFHb and OPG was independent of both parasitaemia and IL-6, consistent with a direct role of CFHb in WPB release.

Second, although we have demonstrated an increase in OPG in severe knowlesi malaria and hypothesise that this is a result of WBP exocytosis, OPG is also expressed in other tissues (such as vascular smooth muscle cells and macrophages). Thus, we cannot confirm that endothelial cells are the source of the increased plasma OPG. However, the consistent finding of increased endothelial activation in severe malaria^10, 27, 54, 55^, the very early increases in plasma OPG observed in other studies^24, 25^, and the concurrent elevation of and correlation with endothelial cell-specific marker angiopoietin-2, suggest that endothelial cells are a likely source of such markedly elevated levels of OPG.

In conclusion, we have demonstrated that intravascular haemolysis is increased in severe knowlesi malaria, and to a greater extent than falciparum malaria. Furthermore, we demonstrate that CFHb is independently associated with angiopoietin-2 and OPG, and that OPG is associated with endothelial cell adhesion molecules and microvascular and endothelial dysfunction, as well as with clinical biomarkers of severity, including lactate and AKI. These findings suggest that haemolysis-mediated endothelial activation and release of WPB constituents, including OPG, is likely a key contributor to end-organ dysfunction in severe knowlesi malaria.

## Materials and methods

### Ethics statement

The study was approved by the Ethics Committees of the Malaysian Ministry of Health and Menzies School of Health Research. Informed written consent was provided by all participating adults, and by the parent or guardian of any participant aged <18 years.

### Study site and patients

Patients were enrolled as part of a prospective observational study of all malaria patients admitted to Queen Elizabeth Hospital, an adult tertiary-referral hospital in Sabah, Malaysia^5, 10^. For the current study, patients enroled between September 2010 and December 2012 were included if they had PCR-confirmed *P. knowlesi* monoinfection, were non-pregnant, ≥12 years old, had no major comorbidities or concurrent illness and were within 18 h of commencing antimalarial treatment. Severe malaria was defined according to modified WHO criteria, as previously described^10^. Renal function was further assessed using the kidney disease: Improving Global Outcomes (KDIGO) criteria for AKI. Using this definition, AKI is defined as an increase in serum creatinine of ≥26.5 µmol/L within 48 h, or to ≥1.5× baseline^56^. Baseline creatinine was estimated using modification of diet in renal disease (MDRD) equation^56^, with an assumed eGFR of 100 mL/min per 1.73 m^2^. Healthy controls were visitors or relatives of malaria patients, with no history of fever in the past 48 h and with blood film negative for malaria parasites.

Standardised history and physical examination were documented. Haematology, biochemistry, acid–base parameters and lactate (by bedside blood analysis; iSTAT system) were obtained on enrolment. Parasite counts were determined by microscopy, and parasite species identified by PCR^57, 58^. Patients with severe disease were treated with intravenous artesunate, while those with non-severe disease received oral artemisinin combination treatment, as previously described^5^.

### Laboratory assays

Venous blood collected in lithium heparin and citrate tubes was centrifuged (including a second high-spin speed for the citrate tube) within 30 min of collection and plasma stored at −70 °C. Plasma CFHb and vWF were measured on the citrated platelet-free plasma by ELISA (Bethyl Laboratories and Biomedica Diagnostics, respectively). Haptoglobin was measured on lithium heparin plasma by ELISA (ICL Laboratories). Plasma concentrations of angiopoietin-2, P-selectin and adhesion molecules ICAM-1 and E-selectin were measured on lithium heparin plasma using quantikine ELISA kits from RnD. OPG was measured on lithium heparin plasma using a duoset ELISA from RnD. IL-6 was measured by flow cytometry (BD cytometric bead array, Becton Dickinson).

### Measurement of endothelial and microvascular function

Endothelial function was measured non-invasively on enrolment using peripheral arterial tonometry (EndoPAT) by the change in digital pulse wave amplitude in response to reactive hyperaemia, giving a reactive hyperaemia peripheral arterial tonometry (RHPAT) index, as previously described^16^. The RHPAT index is at least 50% dependent on endothelial NO production^59^ and has been shown to be L-arginine responsive in falciparum malaria^16^. Measurement of endothelial function was discontinued on patients with non-severe malaria in July 2011. Microvascular function was assessed on enrolment as previously described^60^, using near infra-red spectroscopy (InSpectra 650, Hutchinson Technology, Hutchinson, MN) as previously reported^10^.

### Statistics

Statistical analysis was performed with STATA software (version 14). For continuous variables, intergroup differences were compared using analysis of variance or Kruskal–Wallis tests depending on distribution. Student’s *t*-test or Wilcoxon–Mann–Whitney tests were used for two-group comparisons. Categorical variables were compared using *χ*^2^ or Fisher’s exact tests. Associations between continuous variables were assessed using Spearman’s correlation coefficient. Partial correlation was used to evaluate associations between variables after adjusting for parasitaemia, with non-normally distributed variables log-transformed to normality. Backward stepwise regression was used to evaluate predictors of severe malaria and AKI, with variables removed at a significance level of >0.05. For this analysis, patients with hyperparasitaemia as a sole severity criterion were reclassified as having non-severe malaria. For comparison of intravascular haemolysis in *P. knowlesi* vs. *P. falciparum* malaria, median plasma CFHb in patients with severe knowlesi malaria was compared to previously published CFHb measurements from patients with severe falciparum malaria enroled contemporaneously in the same study cohort^30^.

### Data availability

Data will be available on request from the corresponding author.

## Electronic supplementary material

Supplementary Tables
