# Supplementary material for: Intravascular haemolysis in severe Plasmodium knowlesi malaria: association with endothelial activation, microvascular dysfunction, and acute kidney injury
Source: Emerg Microbes Infect. 2018 Jun 6;7:106. doi: 10.1038/s41426-018-0105-2 (PMC5988665; doi:10.1038/s41426-018-0105-2)
Supplement: Supplementary file 1 — Supplementary Tables [file 41426_2018_105_MOESM1_ESM.docx]

**Supplementary Table 1. Cell-free haemoglobin and haptoglobin in patients with severe and non-severe knowlesi malaria, enrolled prior to or following antimalarial treatment.**

|  | Enrolled prior to commencing treatment | Enrolled post-commencing treatment | P value |
| --- | --- | --- | --- |
| Cell-free haemoglobin |  |  |  |
| Non-severe knowlesi malaria | 30,174 (14,698 – 49,656)  n=38 | 39,761 (18,806 – 57,329)  n=116 | 0.203 |
| Severe knowlesi malaria | 49,517 (41,517 – 361,946)  n=5 | 70,379 (28,444 – 162,694)  n=43 | 0.879 |
| Haptoglobin: |  |  |  |
| Non-severe knowlesi malaria | 10.80 (0.070 – 1.480)  n=29 | 0.255 (0.070 – 0.820)  n=70 | 0.159 |
| Severe knowlesi malaria | 0.110 (0.035 – 0.260)  n=5 | 0.110 (0.035 – 0.190)  n=42 | 0.958 |

Numbers represent median (IQR).

**Supplementary Table 2. Logistic regression model for predictors of acute kidney injury and severe malaria in knowlesi malaria, with osteoprotegerin removed as a predictor variable**

|  | **Odds Ratio** | **95% Confidence Interval** | **P value** |
| --- | --- | --- | --- |
| **Predictors of AKI:** |  |  |  |
| Log angiopoietin-2 | 6.22 | 3.02 – 12.81 | <0.0001 |
| Age | 1.08 | 1.05 – 1.11 | <0.0001 |
| **Predictor of severe malaria:** |  |  |  |
| Log angiopoietin-2 | 9.59 | 3.97 – 23.17 | <0.0001 |
| Log parasite count | 1.65 | 1.31 – 2.08 | <0.0001 |

Predictor variables for both models included age, parasite count, cell-free haemoglobin, and angiopoietin-2.

AKI = acute kidney injury, as defined by KDIGO.

**Supplementary Table 3. Logistic regression model for predictors of acute kidney injury and severe malaria in knowlesi malaria, with angiopoietin-2 removed as a predictor variable**

|  | **Odds Ratio** | **95% Confidence Interval** | **P value** |
| --- | --- | --- | --- |
| **Predictors of AKI:** |  |  |  |
| Log osteoprotegerin | 3.38 | 1.86 – 6.14 | <0.0001 |
| Age | 1.07 | 1.04 – 1.10 | <0.0001 |
| **Predictor of severe malaria:** |  |  |  |
| Log osteoprotegerin | 5.27 | 2.56 – 10.86 | <0.0001 |
| Log parasite count | 1.55 | 1.22 – 1.98 | <0.0001 |

Predictor variables for both models included age, parasite count, cell-free haemoglobin, and osteoprotegerin.

AKI = acute kidney injury, as defined by KDIGO.
